# Use of National Pneumonia Surveillance to Describe Influenza A(H7N9) Virus Epidemiology, China, 2004–2013

**DOI:** 10.3201/eid1911.130865

**Published:** 2013-11

**Authors:** Nijuan Xiang, Fiona Havers, Tao Chen, Ying Song, Wenxiao Tu, Leilei Li, Yang Cao, Bo Liu, Lei Zhou, Ling Meng, Zhiheng Hong, Rui Wang, Yan Niu, Jianyi Yao, Kaiju Liao, Lianmei Jin, Yanping Zhang, Qun Li, Marc-Alain Widdowson, Zijian Feng

**Affiliations:** Chinese Center for Disease Control and Prevention, Beijing, China (N. Xiang, T. Chen, W. Tu, L. Li, Y. Cao, B. Liu, L. Zhou, L. Meng, Z. Hong, R. Wang, Y. Niu, J. Yao, K. Liao, L. Jin, Y. Zhang, Q. Li, Z. Feng);; Centers for Disease Control and Prevention, Atlanta, Georgia, USA (F. Havers, M.-A. Widdowson);; Centers for Disease Control and Prevention, USA/China, Beijing (Y. Song)

**Keywords:** pneumonia surveillance, influenza A(H7N9) virus, influenza virus, viruses, influenza, epidemiology, China

## Abstract

In mainland China, most avian influenza A(H7N9) cases in the spring of 2013 were reported through the pneumonia of unknown etiology (PUE) surveillance system. To understand the role of possible underreporting and surveillance bias in assessing the epidemiology of subtype H7N9 cases and the effect of live-poultry market closures, we examined all PUE cases reported from 2004 through May 3, 2013. Historically, the PUE system was underused, reporting was inconsistent, and PUE reporting was biased toward A(H7N9)-affected provinces, with sparse data from unaffected provinces; however, we found no evidence that the older ages of persons with A(H7N9) resulted from surveillance bias. The absolute number and the proportion of PUE cases confirmed to be A(H7N9) declined after live-poultry market closures (p<0.001), indicating that market closures might have positively affected outbreak control. In China, PUE surveillance needs to be improved.

Since 2004, the Chinese Center for Disease Control and Prevention (China CDC) has conducted surveillance for pneumonia of unknown etiology (PUE) to facilitate timely detection of novel respiratory pathogens, such as severe acute respiratory syndrome (SARS) and avian influenza. On March 31, 2013, health authorities in China reported the first human infection with avian influenza A(H7N9) virus to the World Health Organization (*1*). In response to the emergence of A(H7N9), China CDC and provincial and local CDCs introduced testing for A(H7N9) virus of all persons with reported PUE. As of May 3, 2013, a total of 127 laboratory-confirmed A(H7N9) cases, resulting in 24 deaths, had been reported from 10 provinces and municipalities in mainland China (hereafter referred to as affected areas). The median age of these case-patients was 62 years; most (71%) were males.

Most confirmed case-patients had severe disease (*2*–*4*), and an analysis of national influenza-like illness surveillance data has not found evidence of widespread A(H7N9)-associated mild illness (*5*). After preliminary epidemiologic and virologic information pointed to live-poultry markets (LPMs) as a possible source of infection (*2*,*4*), retail and wholesale LPMs were closed in several major cities in which A(H7N9) was confirmed, including Shanghai, Nanjing, and Hangzhou. The number of new cases declined in these cities after LPM closures (*6*).

However, these reports of A(H7N9) geographic occurrence, demographic patterns, and effectiveness of control measures depend not only on the number of confirmed A(H7N9) cases but also on surveillance and on reporting and testing patterns. Although the number of cases has been studied at length, reported cases are a function of surveillance, and A(H7N9) reporting and testing patterns have not been examined in detail. We describe the PUE surveillance system in China and analyze the proportion of tested persons who test positive in mainland China for A(H7N9) by province, age, and sex before and after LPM closures to assess the possible role of surveillance bias.

## Methods

### Surveillance for PUE before A(H7N9) Emergence

From 2004 through March 2013, health care facilities of all types in China were required to report any patient who had no clear diagnosis and whose illness met 4 criteria. These criteria were 1) fever (axillary temperature >38^◦^C); 2) radiologic characteristics consistent with pneumonia; 3) reduced or normal leukocyte count or low lymphocyte count during early stages of disease; and 4) worsening of symptoms or no obvious improvement after 3–5 days of standard antimicrobial treatment.

Upper or lower respiratory tract specimens from each patient were tested for influenza A(H5N1) virus and for SARS-coronavirus (SARS-CoV) and, beginning in October 2012, for Middle East respiratory syndrome coronavirus. Some provinces also tested for seasonal influenza A (subtypes H1N1 and H3N2) and, after 2009, pandemic H1N1 2009 and B viruses, but this testing varied by province. If specimens were negative for A(H5N1) and SARS-CoV, no further testing was required. Data were collected on age, sex, location, occupation, and dates of illness duration and on who reported the case.

Cases were reported by clinicians directly to the China Information System for Disease Control and Prevention (CISDCP), the nationally notifiable disease reporting system, through an Internet-based platform. Before China CDC became involved in any response, expert consultation committees were required at the county, prefecture, and provincial levels to determine whether the case was SARS or A(H5N1) on the basis of clinical or laboratory evidence. If SARS and A(H5N1) were excluded and there was no other diagnosis, cases were designated as “disease of other unknown cause,” and no further investigation was conducted. However, for clusters of PUE cases, i.e., >2 PUE cases for which an epidemiologic link was identified, the provincial CDC sent the specimens to China CDC for further testing if the provincial expert consultation committee could not provide a clear diagnosis, and China CDC would guide or become directly involved in the field investigation if needed.

### Surveillance for PUE after A(H7N9) Emergence

In response to the emergence of A(H7N9), 3 key changes in this system were implemented. First, starting on March 31, 2013, all specimens from reported PUE cases were required to be tested not only for influenza A(H5N1) but also for seasonal influenza A, influenza B, and influenza A(H7N9) by real-time reverse transcription PCR (*3*). If a specimen was positive for influenza A but could not be subtyped, further testing would be performed. If test results for both influenza types A and B were negative, specimens would be tested for SARS-CoV and Middle East respiratory syndrome coronavirus. Second, local-level evaluation of cases was streamlined in early April 2013. After cases were reported, specimens were sent directly for testing to local and/or provincial CDCs, bypassing the expert consultation committees. Third, to avoid delay in A(H7N9) diagnosis, the fourth reporting criterion above (antimicrobial treatment failure) was replaced with a requirement that the pneumonia etiology could not be attributed to an alternative clinical or laboratory diagnoses. Clinicians were given flexibility to determine how to interpret this criterion, and specific tests were not specified.

Respiratory specimens collected from patients whose illnesses meet the modified PUE case definition are sent to the local and/or provincial influenza network laboratory for testing for A(H7N9). (The first A[H7N9] case in a province is confirmed by China CDC and subsequent cases by the provincial CDC.) In addition, as of April 5, clinicians could also specify whether a patient had a suspected or confirmed A(H7N9) case by using a separate specific case definition and laboratory evidence of possible A(H7N9) infection (*7*) and reported directly to CISDCP. In this analysis, we focused only on the historical and current performance of the PUE surveillance system.

To better understand testing patterns during the A(H7N9) outbreak, we looked at historical reporting in the PUE surveillance system from January 2004 through March 2013. We also examined all PUE cases reported to China CDC during March 30–May 3, 2013, and calculated the proportion positive for A(H7N9) by province and in different age and sex groups. To assess whether LPM closures helped control the epidemic and to account for any reduction in testing, we examined the number of confirmed A(H7N9) cases and the proportion of PUE case-patients who tested positive for A(H7N9) in the week before and the 2 weeks after LPM closures in Shanghai (population 30.5 million), Nanjing (population 8.2 million), and Hangzhou (population 8.8 million). The LPMs were first closed in these cities on April 6, April 8, and April 15, respectively. At the time the study was conducted, the estimated median incubation period of A(H7N9) infection was 6 days (interquartile range 4–7) (China CDC, unpub. data). We separated postclosure results into those in the first and second weeks after closure in each LPM (1–7 days and 8–14 days, respectively) and compared proportions before and after LPM closure using a χ^2^ test for trend. A Pearson χ^2^ test was used to compare the proportion of men and women who tested positive for A(H7N9), and significance was defined by α<0.05. SPSS software version 19.0 (SPSS, Chicago, IL, USA) was used for statistical analysis.

## Results

**Figure 1:**
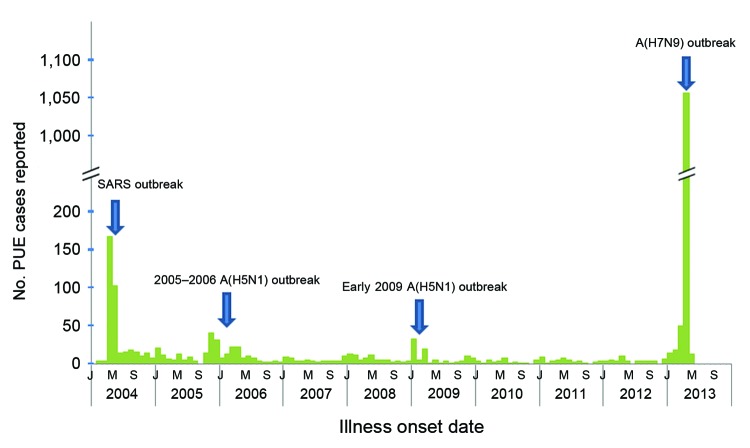
Number of reported PUE cases, mainland China, January 2004–May 2013. SARS, severe acute respiratory syndromes; H5N1, human infection with avian influenza A(H5N1) virus; PUE, pneumonia of unknown etiology.

During January 2004–March 30, 2013, a total of 1,016 cases were reported to the PUE surveillance system, of which 976 (96%) had a final diagnosis available. Thirty-nine (4%) cases were identified as A(H5N1), accounting for 91% of the 43 avian influenza A(H5N1) confirmed in humans in mainland China during 2005–2013. No SARS cases were identified. 744 (76%) PUE cases had no clear final diagnosis. In most months <10 PUE cases were reported, and a mean of 10 cases were reported each month (range 0–168). The number of reported cases increased during identified outbreaks, such as the SARS outbreak in 2004, when the system was first established, and avian influenza A(H5N1) outbreaks in humans during the winter and spring of 2005–06 and early 2009 (Figure 1).

**Figure 2:**
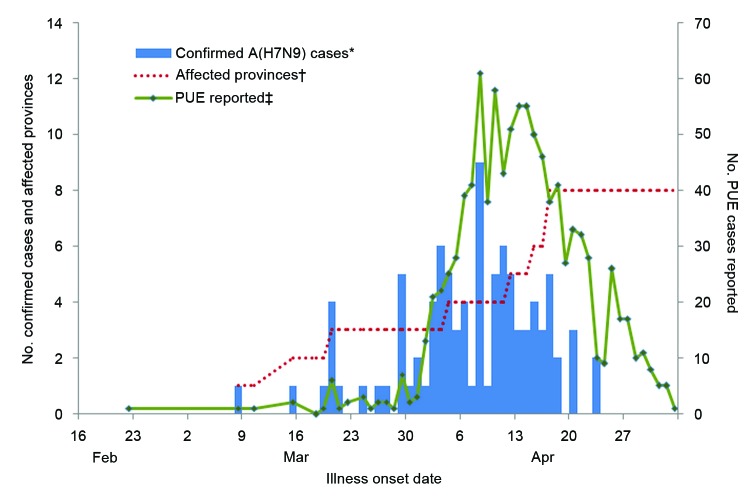
Number of PUE cases, confirmed influenza A(H7N9) cases reported, and cumulative affected provinces or municipalities, mainland China, March 30–May 3, 2013. *Confirmed A(H7N9) cases reported through the PUE surveillance system. †Cumulative affected provinces/municipalities reporting cases through the PUE system. ‡Cases reported through PUE system. PUE, pneumonia of unknown etiology.

During March 30–May 3, 2013, a total of 1,118 PUE cases were reported from 24 provinces, with earliest onset on January 26. PUE cases peaked at 61 per day on April 8, 2013, and then dropped rapidly in the following 3 weeks (Figure 2). A total of 1,002 (90%) PUE cases reported were from affected areas, which constitute 43% of the Chinese population, and 116 (10%) were from from unaffected areas (57% of the population). Most PUE cases were reported from Shanghai (468 [42%] of 1,118) and Zhejiang (388 [26%]). Of the 1,002 PUE cases from affected areas, 94 (9%) were confirmed as A(H7N9), which represents 74% of all 127 confirmed A(H7N9) cases in mainland China as of May 3. The remaining 33 cases were reported either through the influenza-like illness surveillance system (6 cases) or directly to CISDCP (27 cases).

Among the affected areas, Jiangsu reported the highest percentage of PUE positive for A(H7N9) (74%). This was followed by Hunan (33%), Henan (27%), Fujian (18%), Zhejiang (14%), Jiangxi (10%), Shanghai (4%), Beijing (3%), and Anhui and Shandong (0 cases each) (Table 1).

**Table 1:** Numbers of PUE cases and influenza A(H7N9) virus infections reported by PUE surveillance, mainland China, March 30–May 3, 2013*

Province or municipality	No. cases reported	No. (%) A(H7N9) positive
Affected, n = 10		
Anhui	100	0
Beijing	33	1 (3)
Fujian	17	3 (18)
Henan	11	3 (27)
Hunan	6	2 (33)
Jiangsu	27	20 (74)
Jiangxi	42	4 (10)
Shandong	10	0
Shanghai	468	20 (4)
Zhejiang	288	41 (14)
Unaffected, n = 21	116	0
Total	1118	94 (8)

*PUE, pneumonia of unknown etiology.

Of all PUE cases from the affected areas, 288 (29%) occurred in persons <25 years of age; 399 (40%) were 25–59 years, and 315 (31%) were >60 years. The number of PUE cases among female patients was lower overall (449 [45%] of 1,002) and in each age group except the 15–24-year and 25–59-year groups. Among persons >60 years of age, many more men than women were reported through the PUE systems (198 men vs. 117 women) (Table 2).

**Table 2:** Number of reported PUE cases and number positive for influenza A(H7N9) virus in 10 affected areas, mainland China, March 30–May 3, 2013*

Age group, y	Total patients			Male patients			Female patients			p value†
	PUE, no.	A(H7N9) positive, no. (%)		PUE, no.	A (H7N9) positive, no. (%)		PUE, no.	A(H7N9) positive, no. (%)		
<1–4	68	0		44	0		24	0		
5–14	92	1 (1)		52	0		40	1 (3)		0.435
15–24	128	0		64	0		64	0		
25–59	399	42 (11)		195	28 (14)		204	14 (7)		0.015
>60	315	51 (16)		198	34 (17)		117	17 (15)		0.539
Total	1002	94 (9)		553	62 (11)		449	32 (7)		0.027

*PUE, pneumonia of unknown etiology. †p value comparing proportion positive among males with proportion positive among females. Pearson χ2.

Of PUE cases confirmed to be A(H7N9), 1 (1%) was in the 5–14-year age group, 42 (45%) were in patients 25–59 years of age, and 51 (54%) were in patients >60 years of age. The proportion of PUE cases positive for A(H7N9) was higher in adults (11% and 16% in persons 25–59 and >60 years of age, respectively) than in children, teenagers, and young adults (0%, 1%, and 0% in persons <1–4, 5–14, and 15–24 years of age, respectively). Overall, more positive A(H7N9) cases occurred in men than in women (62 vs. 32), and men and women differed significantly in the proportion positive for A(H7N9) (11% vs. 7%, p = 0.027). In persons >60 years of age, twice as many A(H7N9) cases occurred in men than in women (34 vs. 17), although the proportion of PUE cases that were positive for A(H7N9) was not significantly higher in men than in women (17% vs. 15%; p = 0.539) (Table 2).

**Figure 3:**
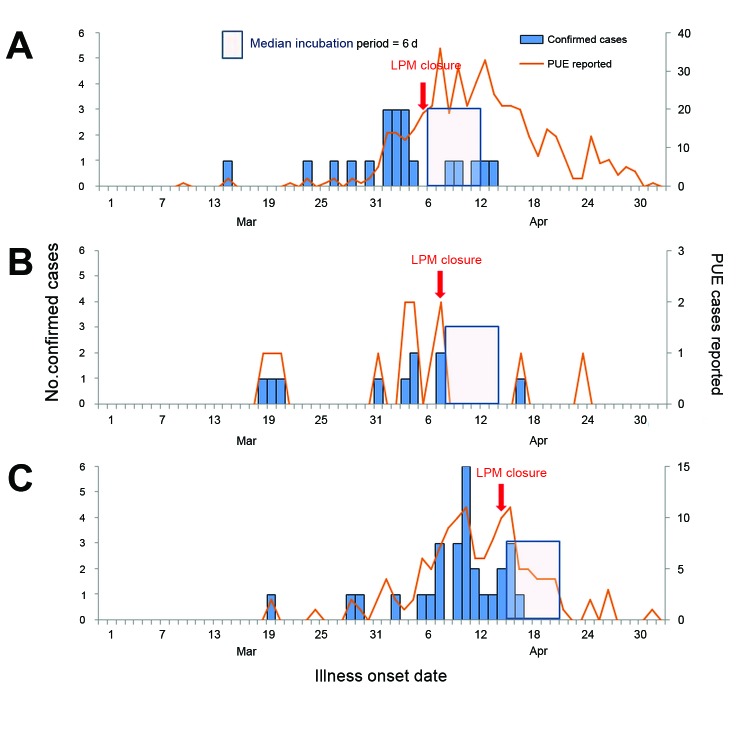
Reported PUE cases and confirmed influenza A(H7N9) cases reported before and after LPM closures, Shanghai (A), Nanjing (B), and Hangzhou (C), mainland China, March 30–May 3, 2013. PUE, pneumonia of unknown etiology; LPM, live-poultry market; J, January; M, May; S, September.

The total number of PUE reported cases declined after LPM closures in Hangzhou and Nanjing but increased in Shanghai in the 1–6 days after closure, then dropped in the 7–14 days after closure. The number of confirmed A(H7N9) cases in Shanghai and Hangzhou after officials closed LPMs declined from 11 and 15 cases, respectively, in the week before closure to 4 and 4 cases during the 1–7 days after closure. In the 8–14 days after closure, 1 and 0 cases were confirmed in those cities, respectively. The proportion of PUE cases positive for A(H7N9) also declined from 14% and 25% before closure to 2% and 12% 1–7 days later and 1% and 0% 8–14 days later, respectively (χ^2^ test for trend, p<0.001 in Shanghai; p = 0.056 in Hangzhou). In Nanjing, 5 positive A(H7N9) cases occurred in the week before LPM closure, with 1 in the 14 days after closure (p = 0.564). When data from the 3 areas are combined, the number of positive cases declined from 31 cases in the week before closure (21% of PUE cases positive for A[H7N9]) to 8 cases (4% positive) 1–7 days after closure; it decreased further to 2 cases (2% positive) in the 8–14 days after closure (p<0.001). In Shanghai, >1.5 times the number of PUE cases were tested for A(H7N9) in the 8–14 days after LPM closure than before closure, although testing decreased in Hangzhou and Nanjing after LPM closure. These data suggest that the decline in absolute numbers was not a surveillance artifact but a real effect (Table 3; Figure 3).

**Table 3:** Reported PUE cases that were positive for influenza A(H7N9) virus before and after closure of live-bird markets in 3 cities, mainland China, March 30–May 3, 2013*

Location	0–6 d before closure†			1–7 d after closure†			8–14 d after closure†		p value
	No. PUE	A(H7N9) positive, no. (%)		No. PUE	A(H7N9) positive, no. (%)		No. PUE	A(H7N9) positive, no. (%)	
Shanghai	81	11 (14)		188	4 (2)		122	1 (1)	<0.001
Nanjing	7	5 (71)		0	0		1	1 (100)	0.564
Hangzhou	60	15 (25)		34	4 (12)		5	0	0.056
Total	148	31 (21)		222	8 (4)		128	2 (2)	<0.001

*PUE, pneumonia of unknown etiology. †Dates of market closures: Shanghai: April 6; Nanjing: April 8; Hangzhou: April 15, 2013. ‡χ^2^ test for trend for percentage of reported cases testing positive for A(H7N9).

## Discussion

Our study examined the Chinese national PUE surveillance system and its utility during the influenza A(H7N9) outbreak in the spring of 2013. Historically, the PUE system had been underused, and reporting had been inconsistent. The number of reported PUE cases increased above minimum levels only during known outbreaks of A(H5N1) and SARS, the only pathogens for which there had been testing. We describe several changes made to the PUE system during the A(H7N9) outbreak that increased its sensitivity and timeliness, resulting in increased reporting; yet, we demonstrated low frequency of PUE reporting from unaffected provinces. Moreover, some provinces were clearly prescreening possible A(H7N9) PUE cases before reporting, which resulted in wide variations in percent positivity. Nevertheless, data from the PUE system demonstrated that 1) A(H7N9) cases were indeed more common in elderly persons; 2) men are at higher risk than women for PUE and A(H7N9) virus infection; and 3) the decline in reported cases after LPM closure probably reflects a true decline in the number of cases, not merely a decline in testing.

Historical data from the PUE surveillance system demonstrated that the system has consistently been underused. Before the A(H7N9) outbreak, it was used to report most A(H5N1) cases in China. However, the PUE system was not (and still is not) used consistently. In 1 study, which examined all cases of community-acquired pneumonia in 6 hospitals over 1 year (April 1, 2008–March 31, 2009), 442 (29%) of the 1,506 community-acquired pneumonia cases met PUE criteria and should have been reported to the PUE system (*8*). In contrast, only 1,016 PUE cases in all of China were reported during a 9-year period. We showed that the number of cases surged when an outbreak occurred, either during the SARS outbreak or during publicized A(H5N1) outbreaks. This surge may reflect enhanced administrative requirements from health authorities (*9*) or enhanced clinician awareness of respiratory viruses.

Before April 2013, the administrative burden of reporting a case to the PUE system gave clinicians little incentive to participate. Reporting a PUE case triggered requirements, such as cooperating with an epidemiologic investigation, collecting specimens, providing clinical information for expert committees, and moving patients to isolation wards. In return, clinicians received little information; 76% of reported PUE cases had no final specific diagnosis, and clinicians were told only whether the cases were SARS or A(H5N1). Streamlining the PUE reporting system and decreasing the requirements involving expert consultation committees probably contributed to the large increase in PUE reporting during the A(H7N9) outbreak; more PUE cases were reported during the study period than in the prior 9 years of PUE surveillance.

During the A(H7N9) epidemic, reporting increased substantially only in affected areas, leading to huge variation between provinces in PUE reporting. Of most concern is that during the A(H7N9) outbreak, areas with no human cases grossly underreported PUE cases. Most (92%) reported PUE cases were negative for A(H7N9) and were probably caused by other etiologies. Thus, we would expect to see a comparable number of PUE cases reported in affected and unaffected areas. However, 68% of all PUE cases were reported from Shanghai and Zhejiang province; together, these 2 provinces constitute only 6% of the total population of China. By contrast, only 10% of all PUE cases were reported in the 21 unaffected provinces; these constitute 57% of the population (*10*).

In addition to surveillance bias away from provinces unaffected by the A(H7N9) outbreak, variation probably occurs among provinces in the screening that precedes reporting a PUE case. Some provinces reported PUE cases before extensive testing; in other provinces, clinicians may send specimens directly to the local CDC for testing first, then report only those that had a positive result as PUE cases. This scenario was documented in a previous analysis of the PUE system during 2004–2007 (*11*). The discrepancy in the proportion of positive cases in different provinces (74% in Jiangsu vs. 4% in Shanghai) indicates that prescreening was most likely a factor in PUE reporting practices during the A(H7N9) outbreak. The sharp decline in PUE reporting noted after mid-April also might reflect increased availability of A(H7N9) testing at the local and provincial levels. The ability to test for A(H7N9) locally enables clinicians and local health officials to bypass PUE reporting and instead report a case to CIDSP as a suspected or confirmed A(H7N9) case; this raises the question of how much the PUE system will be used if future large outbreaks of A(H7N9) occur.

Despite the limitations of the PUE reporting system, it yielded important epidemiologic information. First, we found that the older age distribution of persons with A(H7N9) was probably true and not a result of surveillance bias because testing was extensive among young persons, and the percentage positive increased in persons >60 years of age. This contrasts sharply with A(H5N1) cases in China in which the median age of infection is 26 years (*12*). Second, more PUE cases were reported among men who were also more likely to test positive; the reason may be that men are at higher risk for any pneumonia, perhaps because of underlying respiratory comorbidities, but the increased percentage positive for A(H7N9) among men also suggests a specific risk for A(H7N9), especially among working-aged men. The reason may be that these men are more exposed to poultry through occupation or behavior. Third, PUE surveillance analysis suggested that LPM closure did reduce A(H7N9) transmission to humans, whereas a previous report indicated that the number of new A(H7N9) cases declined after LPM closure (*6*), this decline could have reflected decreased testing and not an actual decline in A(H7N9) incidence. Our analysis shows that, although the number of persons reported with PUE and tested for A(H7N9) virus decreased after LPM closure, the proportion of PUE testing positive for A(H7N9) also decreased in the weeks after closure. Investigation of A(H7N9) cases in China has found that 77% of cases for which information was available have had poultry exposure, many through contact with LPMs (*2*). In the 1997 outbreak of A(H5N1) in Hong Kong, poultry were culled and LPMs closed (*13*). These measures controlled the outbreak, and A(H5N1) disease was not reported again in humans until 2003.

Our study has several limitations. First, the incidence of A(H7N9) in the 3 areas with LPM closure that we studied may have decreased regardless of LPM closure. It is possible that LPM closures were associated with—but not the cause of—the waning number of cases. This decreasing incidence could have been the case had there been a short wave of infected poultry passing through LPMs. Also possible is that, as with A(H5N1), A(H7N9) may be seasonal in birds and therefore in humans, with lower transmission during the spring and summer months. Second, although we demonstrate that the proportion of PUE cases positive for A(H7N9) decreased after LPM closure, the substantial decrease in reporting and testing immediately after market closure in Hangzhou may have resulted in missed cases and exaggerated the apparent effect of closure. In addition, how much increased local testing for A(H7N9) may have affected PUE reporting is unknown.

This study identified several major problems with the PUE surveillance system, including low and uneven levels of participation and inconsistency among provinces in how the system is used. Given its potential value in monitoring future A(H7N9) activity, the system’s overall objectives and reporting procedures should be further evaluated. The continued threat of additional viral adaptation to human hosts leading to increased transmissibility lends added urgency to the ongoing improvement of the PUE system to better understand the epidemiology of A(H7N9), detect outbreaks, and evaluate control measures.
